# Genetic Analysis of the Relationship between Bone Mineral Density and Low-Density Lipoprotein Receptor-Related Protein 5 Gene Polymorphisms

**DOI:** 10.1371/journal.pone.0085052

**Published:** 2013-12-23

**Authors:** Jiayong Yi, Yu Cai, Zhenjun Yao, Jianping Lin

**Affiliations:** 1 Department of Orthopedics, Zhongshan Hospital, Fudan University, Shanghai, P. R. China; 2 Department of Gastroenterology, Zhongshan Hospital, Fudan University, Shanghai, P. R. China; Inserm U606 and University Paris Diderot, France

## Abstract

**Background:**

A number of studies have examined the association between the polymorphisms of the low-density lipoprotein receptor-related protein 5 gene (LRP5), but previous results have been inconclusive. Thus we performed a meta-analysis of studies on the association between the LRP5 polymorphisms and bone mineral density (BMD) to assess their pooled effects.

**Methods:**

Published literature from PubMed, EMBASE and ISI web of science were searched for eligible publications. Weighted mean difference (WMD) and 95% confidence interval (CI) was calculated using fixed- or random-effects model.

**Results:**

A total of 19 studies with 25773 subjects were considered in this meta-analysis. Of them, 17 examined the association between the A1330V polymorphism and BMD, 8 were focused on the V667M polymorphism, and 2 analyzed the Q89R polymorphism. Individuals with the A1330V AA genotype showed significantly higher BMD than those with the AV/VV genotypes [at lumbar spine (LS): WMD = 0.02g/cm^2^, 95% CI = 0.01-0.03, *P* < 10^-4^; at femur neck (FN): WMD = 0.01g/cm^2^, 95% CI = 0.00-0.02, *P* = 0.01] or VV genotype (at LS: WMD = 0.02g/cm^2^, 95% CI = 0.01-0.04, *P* = 0.01). Significant associations were also detected in the analysis for V667M (VV vs. VM/MM: WMD at LS = 0.02g/cm^2^, 95% CI = 0.02-0.03, *P* < 10^-5^; WMD at FN = 0.01g/cm^2^, 95% CI = 0.01-0.02, *P* = 0.0002). As for Q89R, subjects with the QQ genotype tended to have higher BMD than those with the QR/RR genotypes at FN (WMD = 0.03g/cm^2^, 95% CI = 0.01-0.05, *P* = 0.005).

**Conclusion:**

This meta-analysis demonstrated that the *LRP5* polymorphisms may be modestly associated with BMD of LS and FN.

## Introduction

Osteoporosis is a common disease characterized by low bone mass, microarchitectural deterioration of bone tissue and enhanced bone fragility which leads to an increased incidence of fracture. It is now well established that genetic factors play a major role in regulating bone mineral density (BMD) [1]. Association studies have been used to identify genetic variants that are associated with BMD and fractures [2–6]. Recently, genome-wide association studies (GWAS) have been successful in further identifying several common variants that are significantly associated with BMD and with fracture risk [7–10].

The low-density lipoprotein receptor (LDLR)-related protein 5 (*LRP5*) gene is one of the candidate genes that have been implicated in BMD. As a member of the LDLR family, this gene contains 23 exons that span more than 100 kb and encodes a single-pass transmembrane protein of 1614 amino acids [11]. LRP5 cooperates with members of the frizzled family of seven-pass transmembrane receptors to bind Wnt proteins, and forms a functional ligand-receptor complex that activates the Wnt-β-catenin pathway [12–14]. This Wnt-β-catenin pathway is one of the key pathways to affect osteoblast development. It has been reported that loss-of-function mutations of the *LRP5* gene cause osteoporosis-pseudoglioma (OPPG), an autosomal recessive disease characterized by low bone mass and childhood fractures [15], whereas the *LRP5* G171V mutation is associated with autosomal dominant high bone mass (HBM) traits [16–18]. Transgenic and knock-out mouse models mimic the human phenotypes of high and low bone mass, respectively [19]. In addition to these mutations, a number of polymorphisms have been described in the *LRP5* gene. Among them, two coding polymorphisms A1330V (rs3736228) and V667M (rs4988321) were studied most, and were suggested to be associated with BMD [6,20–24]. Another missense variant is Q89R (rs41494349) located in exon 2, which has been reported to be significantly associated with BMD among Asian population [25,26].

Although many studies have investigated the relationship between these three *LRP5* polymorphisms and BMD, published results have been conflicting. This may be due to that individual studies based on restricted sample sizes lack sufficient statistical power to detect effects of interest. Therefore, we performed the present meta-analysis to clarify the association of the *LRP5* polymorphisms (A1330V, V667M and Q89R) with BMD.

## Materials and Methods

### Literature search strategy

The literature included in our analysis was selected from PubMed, EMBASE and ISI web of science using combinations of the following keywords: low-density lipoprotein receptor-related protein 5 gene (*LRP5*), polymorphism, A1330V (rs3736228), V667M (rs4988321), Q89R (rs41494349) and bone mineral density (BMD). Genetic association studies published before the end of March 1, 2013 on the association between BMD and polymorphisms in the *LRP5* gene were retrieved, and their references were checked to identify other relevant publications. The publication language was restricted to English. 

### Eligible studies and data extraction

Eligible studies had to meet all of the following criteria: (1) were published in peer-reviewed journals and were independent studies using original data; (2) investigated the effect of the *LRP5* polymorphisms on BMD; (3) provided sufficient data for calculation of weighted mean difference (WMD) with its 95 % confidence interval (CI) and *P* value; (4) described the genotyping method, equipment, and protocols used or provided reference to them. For each study, the following data were extracted independently by two authors: first author’s surname, year of publication, age, gender, ethnicity, study site, genotyping method, total number of subjects, skeletal sites evaluated for BMD, and genotype and BMD data. The results were compared and disagreements were discussed and resolved with consensus. Studies with different gender, age and ethnic groups were considered as individual studies for our analyses.

### Statistical analysis

We pooled eligible studies according to the site of BMD measurement and performed analyses at the lumbar spine (LS), total hip (HT), femoral neck (FN) and trochanter, respectively. The main analyses addressed differences in BMD between genotypes: AA versus AV/VV or AA versus VV for A1330V; VV versus VM/MM for Val667Met; QQ versus QR/RR for Q89R. We calculated the weighted mean difference (WMD) based on the actual BMD values reported in the included studies. Cochran’s Chi square-based Q statistic test was performed to assess possible heterogeneity between the individual studies [27]. If heterogeneity existed (*P* < 0.05) across studies, the random effects model was used [28]; otherwise, the fixed effect model was adopted [29]. We also performed subgroup analyses based on ethnicity (Caucasian and Asian) and gender (women and men).

Sensitivity analyses were performed to assess the stability of the results, namely, each study in the meta-analysis was deleted each time to reflect the influence of the individual dataset to the overall OR. Publication bias was assessed by Egger’s test [30] and funnel-plot analysis. All *P* values are two-sided, and *P* < 0.05 was considered statistically significant. All the statistical analyses were performed by Review Manager v.5.

## Results

### Characteristics of studies

**Figure 1:**
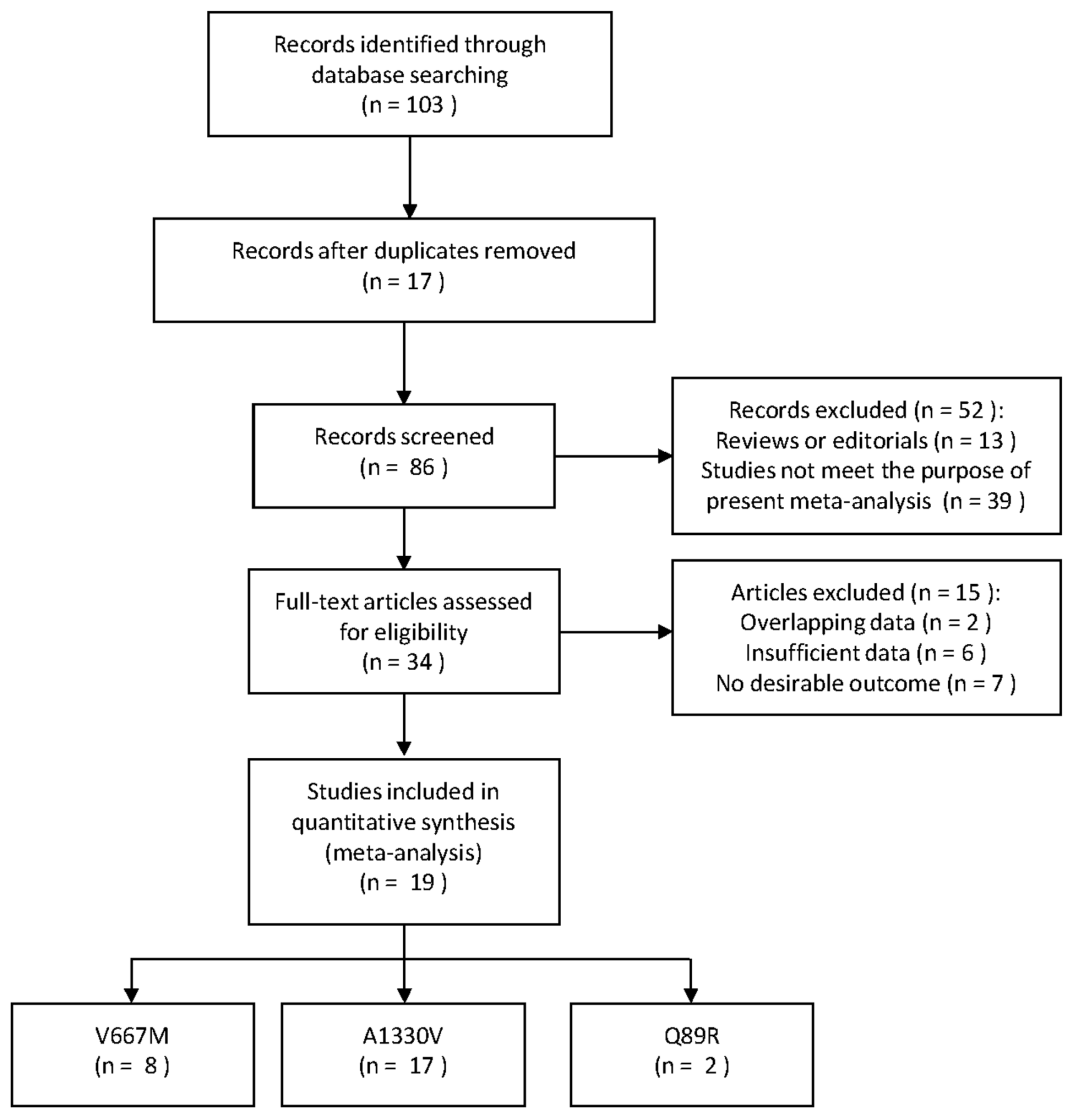
Summary of search strategy and result.

The literature search yielded 103 references. Study selection process is shown in Figure 1 and Table S1. A total of 19 eligible studies [20-26,31-42] were finally included with 25773 subjects (15871 women and 9902 men). For the A1330V polymorphism, 17 studies were available, including a total of 23837 subjects. For the V667M polymorphism, 8 studies involved a total of 14985 Caucasian subjects. For the Q89R polymorphism, 2 studies with a total of 1069 subjects were included, both of which were conducted in Asia. The detailed characteristics of all the studies and the main results in this meta-analysis are shown in Tables 1, 2, 3 and 4.

**Table 1:** Characteristics of individual studies included in the meta-analysis.

Author	Year	Gender	Country	Ethnicity	Age (mean ± SD)	Genotyping method	Number	BMD	SNP
Brixen[21]	2007	Men	Denmark	Caucasian	20-30	TaqMan/Acyclo Prime-FP SNP Detection kits	783	LS, TH	Ala1330Val
Ezura[23]	2007	Women	Japan	Asian	64.6±10.8	Sd-PCR/Invader assay/TaqMan	387	LS	A1330V
Giroux[24]	2007	Women	Canada	Caucasian	25-91	Allele-specific PCR	5144	LS, FN	Val667Met, Ala1330Val
Giroux[31]	2008	Women	Canada	Caucasian	18-58	Allele-specific PCR	1382	LS, FN	Val667Met
Grundberg [32]	2008	Men	Sweden	Caucasian	Sweden 74.4±3.2； Hong Kong 72.4±5.0；GOOD 18.9±0.56	TaqMan	6082	LS, FN	Val667Met, Ala1330Val
Jiang[33]	2010	Men and women	China	Asian	48.9±12.5	TaqMan	425	LS, TH	A1330V
Koh[25]	2004	Men	Korea	Asian	25.6±3.7	PCR-RFLP	219	LS, FN, trochanter	Q89R, A1330V
Koller[34]	2005	Women	USA	Caucasian	33.3±7.0	Fluorescent allele-specific PCR/MALDI-TOF	1301	LS, FN	A1330V
Kruk[35]	2009	Men	UK	Caucasian	59.7±10.7	PCR-Sequencing	249	LS, TH, FN, trochanter	Val667Met, Ala1330Val
Markatseli [36]	2011	Women	Greece	Caucasian	56.8±4.9	TaqMan	221	LS	Val667Met, Ala1330Val
Massart[37]	2012	Women	Italy	Caucasian	20-50	PCR-RFLP	570	LS, TH, FN	Val667Met, Ala1330Val
Mencej- Bedrac[38]	2009	Men and women	Slovenia	Caucasian	62.3±9.5	PCR-RFLP	625	LS, FN	A1330V
Meurs[22]	2006	Men and women	Netherlands	Caucasian	>50	PCR-SBE	5373	LS, FN	A1330V
Mizuguchi [39]	2004	Women	Japan	Asian	54.2±12.4	PCR-Sequencing/TaqMan	254	LS	A1330V
Riancho[42]	2011	Women	Spain	Caucasian	51-90	Mass-array/ TaqMan	873	FN	A1330V
Saarinen[20]	2007	Men	Finland	Caucasian	18-21	PCR-Sequencing	235	LS, TH, FN, trochanter	A1330V
Stathopoulou[40]	2010	Women	Greece	Caucasian	>50	iPLEX Gold assay	554	LS, TH, FN, trochanter	Val667Met
Yu[41]	2010	Men	China	Asian	30.6±6.3	PCR-RFLP	422	LS, TH, FN, trochanter	A1330V
Zhang[26]	2005	Women	China	Asian	60.1±6.3	PCR-RFLP	647	LS, FN, trochanter	Q89R, A1330V

BMD: bone mineral density; TH: total hip;LS: lumbar spine; FN: femur neck.

**Table 2:** Meta-analysis of the A1330V polymorphism and BMD association.

Site of BMD	LS				TH			
Subgroup	No. of data sets	WMD [95% CI]	*P* value	*I* ^2^(*P*)	No. of data sets	WMD [95% CI]	*P* value	*I* ^2^(*P*)
Overall								
AA vs AV/VV	11	0.02 [0.01,0.03]	**<10^-4^**	51% (0.02)	2	0.03 [-0.07,0.14]	0.53	91% (0.001)
AA vs VV	11	0.02 [0.01,0.04]	**0.01**	6% (0.39)	7	0.00 [-0.05,0.05]	1.00	67% (0.006)
Ethnicity								
Caucasian	7	0.02 [0.01,0.03]	**0.001**	67% (0.005)	5	-0.04 [-0.08,0.00]	0.05	0% (0.42)
Asian	4	0.01 [0.00,0.02]	**0.04**	0% (0.68)	2	0.07 [-0.01,0.15]	0.07	64% (0.09)
Gender								
Women	5	0.03 [0.01,0.04]	**0.0003**	65% (0.02)	3	-0.06 [-0.13,0.01]	0.11	0% (0.65)
Men	6	0.01 [0.00,0.02]	**0.04**	0% (0.47)	3	0.00 [-0.07,0.06]	0.89	69% (0.04)
Site of BMD	FN				Trochanter			
Subgroup	No. of data sets	WMD [95% CI]	*P* value	*I* ^2^(*P*)	No. of data sets	WMD [95% CI]	*P* value	*I* ^2^(*P*)
Overall								
AA vs AV/VV	8	0.01 [0.00,0.02]	**0.01**	52% (0.04)	3	-0.00 [-0.01,0.02]	0.59	83% (0.003)
AA vs VV	9	0.00 [-0.01,0.02]	0.67	24% (0.23)	2	-0.03 [-0.13,0.09]	0.76	85% (0.009)
Ethnicity								
Caucasian	6	0.01 [0.01,0.02]	**0.01**	56% (0.04)	2	-0.01 [-0.03,0.01]	0.47	90% (0.001)
Asian	2	0.00 [-0.01,0.01]	0.60	0% (0.36)	1	0.01 [-0.01,0.03]	0.22	NA
Gender								
Women	3	0.01 [0.01,0.02]	**<10^-4^**	0% (0.39)	1	0.01 [-0.01,0.03]	0.22	NA
Men	5	0.01 [-0.00,0.01]	0.14	62% (0.03)	2	-0.01 [-0.03,0.01]	0.47	90% (0.001)

**Table 3:** Meta-analysis of the V667M polymorphism and BMD association.

Site of BMD	LS				TH			
Subgroup	No. of data sets	WMD [95% CI]	*P* value	*I* ^2^(*P*)	No. of data sets	WMD [95% CI]	*P* value	*I* ^2^(*P*)
Overall								
VV vs VM/MM	10	0.02 [0.02,0.03]	**<10^-5^**	19% (0.27)	5	0.01 [-0.01,0.02]	0.43	23% (0.27)
Ethnicity								
Caucasian	10	0.02 [0.02,0.03]	**<10^-5^**	19% (0.27)	5	0.01 [-0.01,0.02]	0.43	23% (0.27)
Gender								
Women	7	0.03 [0.02,0.03]	**<10^-5^**	39% (0.13)	4	0.01 [-0.01,0.03]	0.32	37% (0.19)
Men	3	0.02 [0.00,0.04]	**0.01**	0% (0.61)	1	0.00 [-0.04,0.3]	0.87	NA
Site of BMD	FN				Trochanter			
Subgroup	No. of data sets	WMD [95% CI]	*P* value	*I* ^2^(*P*)	No. of data sets	WMD [95% CI]	*P* value	*I* ^2^(*P*)
Overall								
VV vs VM/MM	9	0.01 [0.01,0.02]	**0.0002**	42% (0.09)	2	0.01 [-0.01,0.03]	0.31	0% (0.34)
Ethnicity								
Caucasian	9	0.01 [0.01,0.02]	**0.0002**	42% (0.09)	2	0.01 [-0.01,0.03]	0.31	0% (0.34)
Gender								
Women	6	0.02 [0.01,0.02]	**<10^-4^**	52% (0.06)	1	0.02 [-0.01,0.05]	0.16	NA
Men	3	0.00 [-0.01,0.02]	0.58	0% (0.51)	1	-0.00 [-0.04,0.03]	0.91	NA

**Table 4:** Meta-analysis of the Q89R polymorphism and BMD association.

Site of BMD	LS				FN				
Subgroup	No. of data sets	WMD [95% CI]	*P* value	*I* ^2^(*P*)	No. of data sets	WMD [95% CI]		*P* value	*I* ^2^(*P*)
Overall									
QQ vs QR/RR	2	0.00 [-0.02,0.03]	0.89	0% (0.86)	2	0.03 [0.01,0.05]		**0.005**	72% (0.06)
Ethnicity									
Asian	2	0.00 [-0.02,0.03]	0.89	0% (0.86)	2	0.03 [0.01,0.05]		**0.005**	72% (0.06)
Gender									
Women	1	0.00 [-0.03,0.03]	0.83	NA	1	0.02 [-0.00,0.04]		0.1	NA
Men	1	0.00 [-0.05,0.04]	0.93	NA	1	0.06 [0.02,0.10]		**0.003**	NA
Site of BMD	Trochanter								
Subgroup	No. of data sets		WMD [95% CI]		*P* value		*I* ^2^(*P*)		
Overall									
QQ vs QR/RR	2		0.02 [-0.00,0.03]		0.10		23% (0.25)		
Ethnicity									
Caucasian	2		0.02 [-0.00,0.03]		0.10		23% (0.25)		
Gender									
Women	1		0.01 [-0.01,0.03]		0.26		NA		
Men	1		0.04 [-0.01,0.09]		0.10		NA		

### Association of the LRP5 A1330V polymorphism with BMD

**Figure 2:**
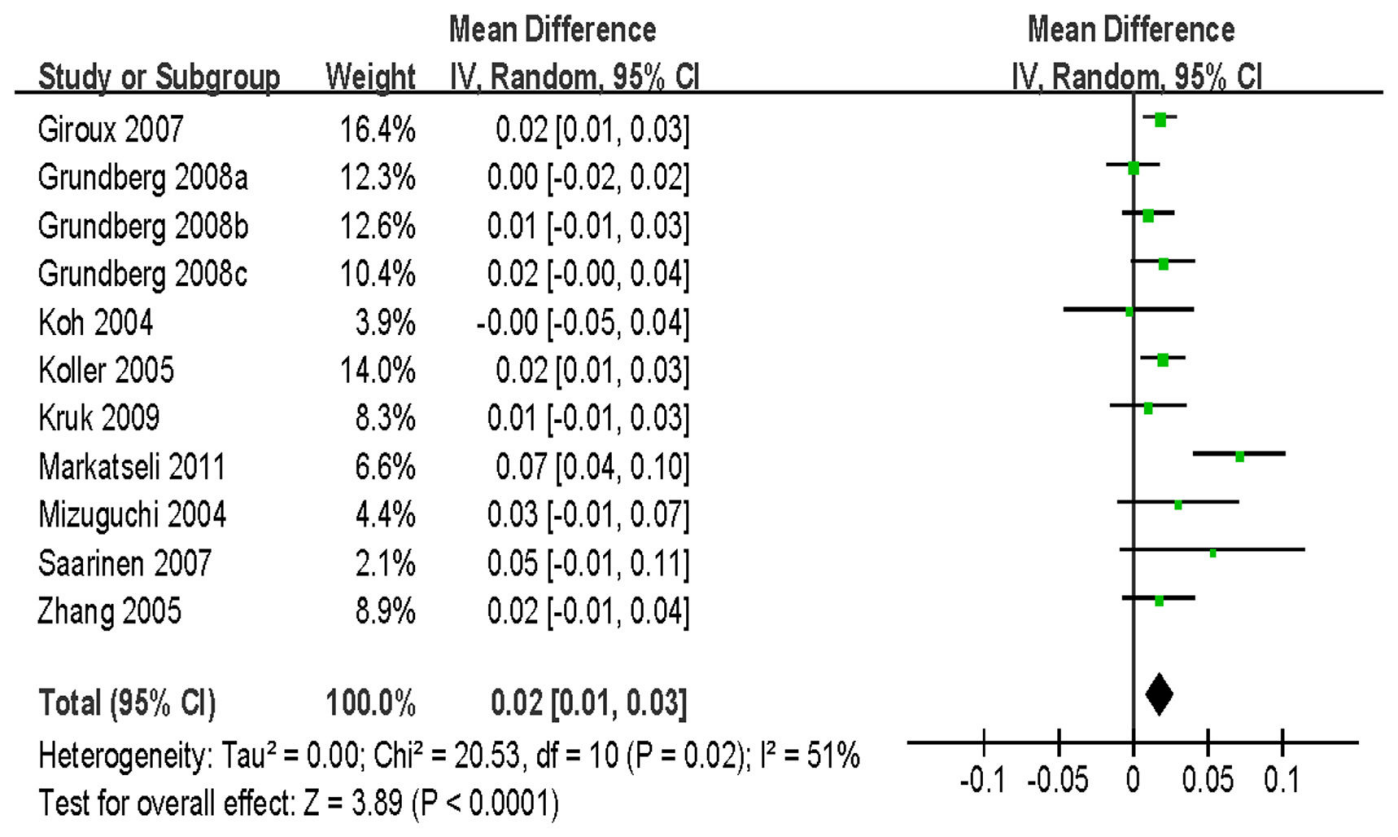
WMD and 95% CI in LS BMD between A1330V AA and AV/VV genotypes.

**Figure 3:**
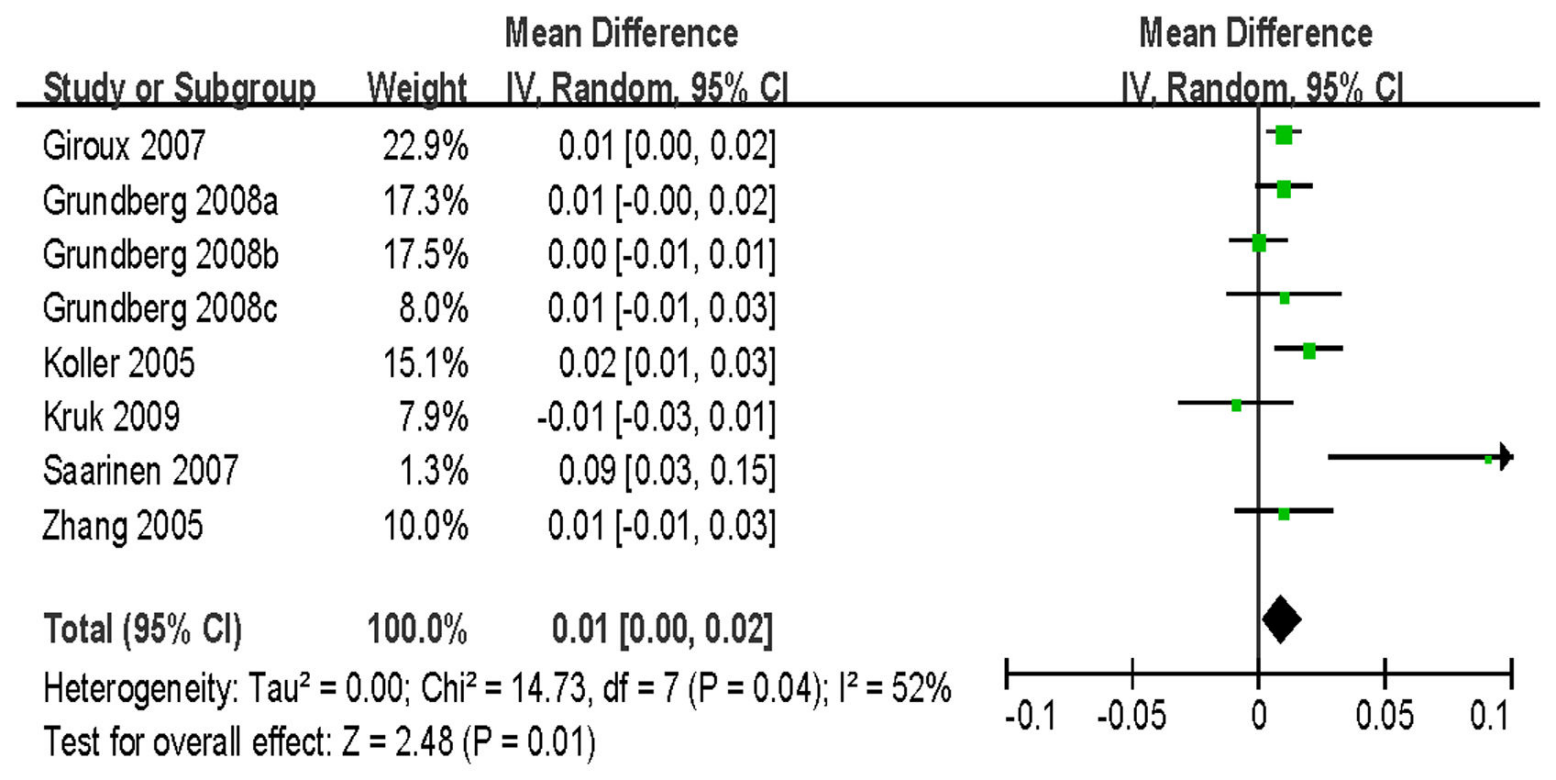
WMD and 95% CI in FN BMD between A1330V AA and AV/VV genotypes.

There were 17 studies analyzing LS BMD, 6 analyzing TH BMD, 12 analyzing FN BMD and 5 focused on trochanter BMD. In the pooled analyses, individuals with the AA genotype were found to have a statistically significant association with BMD compared to subjects with the AV/VV (at LS: WMD = 0.02 g/cm^2^, 95% CI = 0.01-0.03, *P* < 10^-4^; at FN: WMD = 0.01 g/cm^2^, 95% CI = 0.00-0.02, *P* = 0.01) (Figure 2 and Figure 3) or VV genotype (at LS: WMD = 0.02 g/cm^2^, 95% CI = 0.01-0.04, *P* = 0.01). Similar results were obtained when stratified by gender and ethnicity for AA versus AV/VV (Table 2). Interestingly, the association was not found in men [WMD = 0.01 g/cm^2^, 95% CI = (-0.00)-0.01, *P* = 0.14] or Asians [WMD = 0.00 g/cm^2^, 95% CI = (-0.01)-0.01, *P* = 0.60] in the analysis of FN BMD. We did not observe any significant association for BMD of TH and trochanter (Table 2).

### Association of the LRP5 V667M polymorphism with BMD

**Figure 4:**
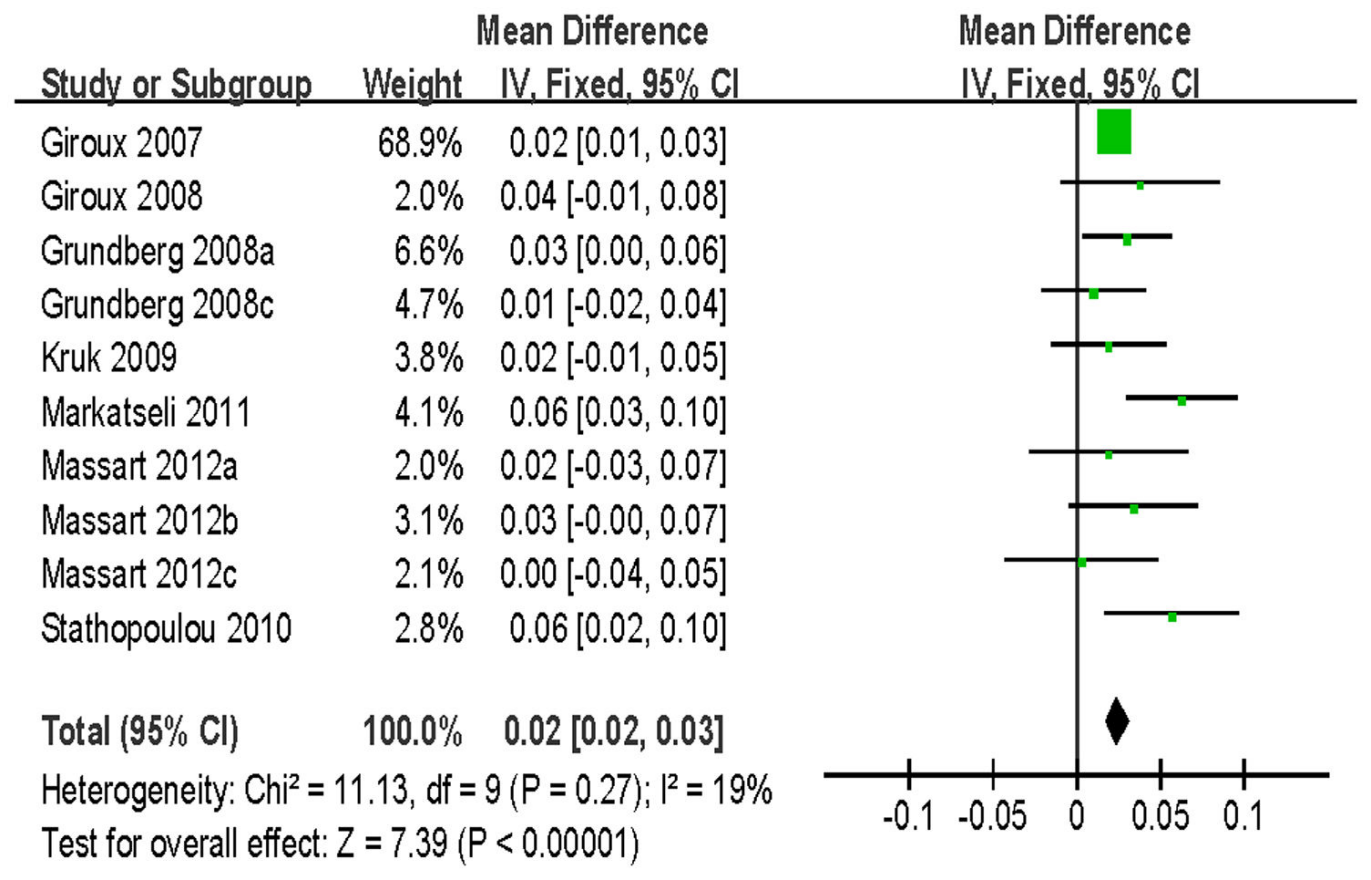
WMD and 95% CI in LS BMD between V667M VV and VM/MM genotypes.

**Figure 5:**
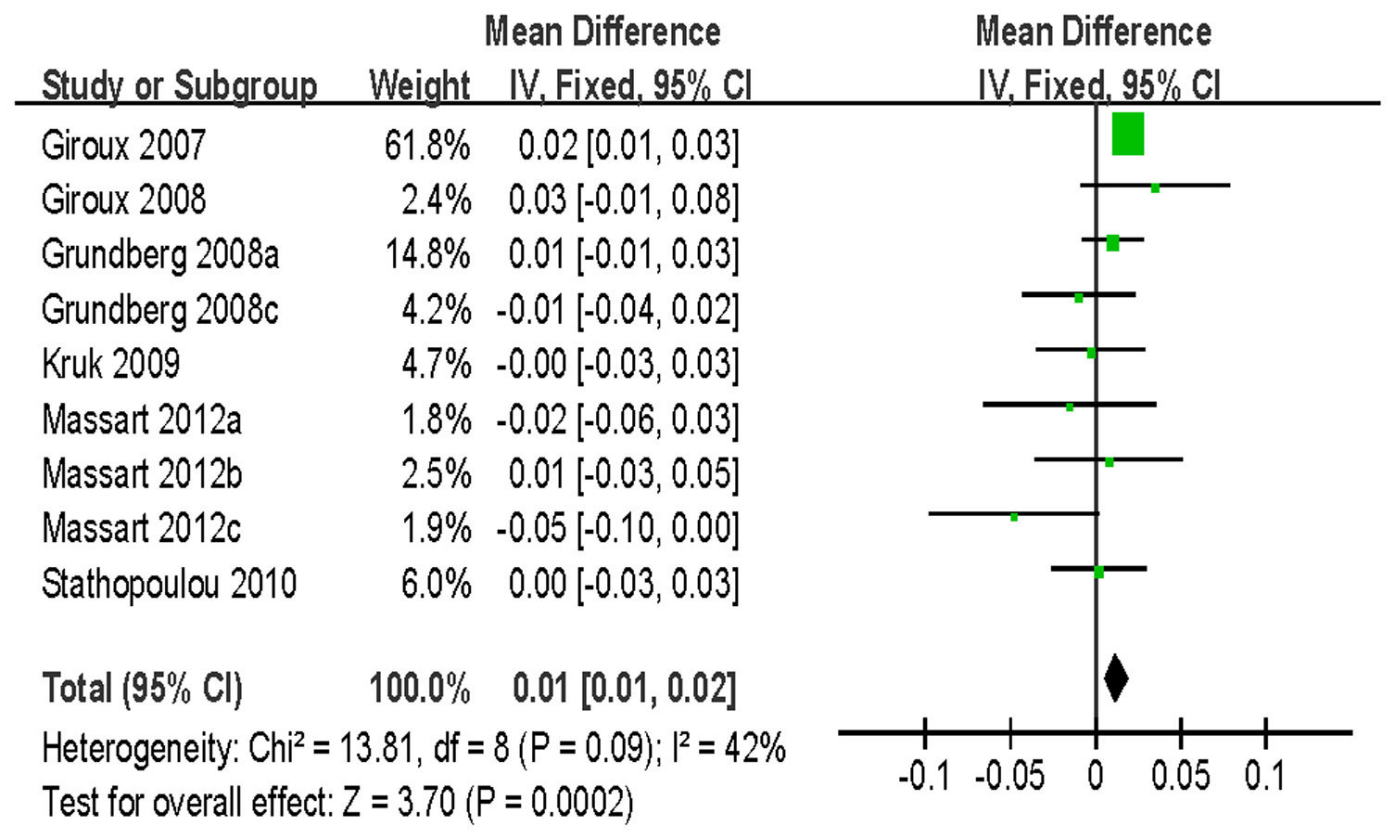
WMD and 95% CI in FN BMD between V667M VV and VM/MM genotypes.

For BMD of LS, 7 studies with 8242 women and 4323 men were identified for the data analysis. Individuals with the VV genotype showed a significantly greater BMD than those with the VM/MM genotype (WMD = 0.02 g/cm^2^, 95% CI = 0.02–0.03, *P* < 10^-5^) without between-study heterogeneity (*P* = 0.27) (Figure 4). Similar significant associations were observed when women and men genotypes were analyzed separately (Table 3). For BMD of FN, a total of 10973 subjects based on 6 studies were identified for the data analysis. There was a significant association detected between the polymorphism and BMD (VV vs. VM/MM: WMD = 0.01 g/cm^2^, 95% CI = 0.01–0.02, *P* = 0.0002) (Figure 5). However, no association was found for men in the subgroup analysis stratified by gender [VV vs. VM/MM: WMD = 0.00 g/cm^2^, 95% CI = (-0.01)-0.02, *P* = 0.58]. Furthermore, our data showed no statistical evidence of significant association between this SNP and BMD of TH and trochanter (Table 3).

### Association of the LRP5 Q89R polymorphism with BMD

**Figure 6:**
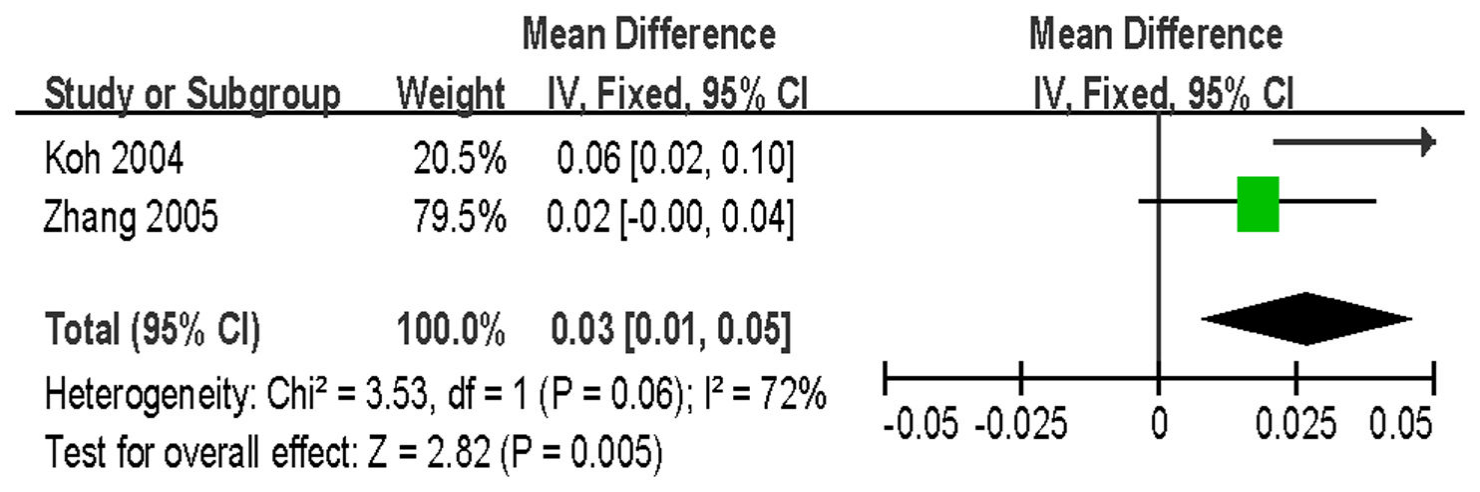
WMD and 95% CI in FN BMD between Q89R QQ and QR/RR genotypes.

Two studies analyzed the relationship between Q89R and the BMD of LS, FN and trochanter among a total of 1069 Asian subjects. There was a significant association between the *LRP5* Q89R polymorphism and FN BMD (QQ vs. QR/RR: WMD = 0.03 g/cm^2^, 95% CI = 0.01–0.05, *P* = 0.005) without between-study heterogeneity (*P* = 0.06) (Figure 6). When stratified by gender, significant association was found only in men (QQ vs. QR/RR: WMD = 0.06 g/cm^2^, 95% CI = 0.02–0.10, *P* = 0.003), but not in women (Table 4). The results of our meta-analyses for LS and trochanter BMD indicated no significant associations [LS: WMD = 0.00 g/cm^2^, 95% CI = (-0.02)-0.03, *P* = 0.89; trochanter: WMD = 0.02 g/cm^2^, 95% CI = (-0.00)-0.03, *P* = 0.10].

### Sensitivity analysis and potential publication bias

Sensitivity analysis was conducted by deleting each study at a time to reflect the influence of the individual dataset to the pooled WMDs, and the corresponding results were not qualitatively altered. The inverted funnel plots were symmetrical as shown in Figures S1-S5. Egger’s test was also performed to access the publication bias of the literatures, and no publication bias was detected for the associations of the *LRP5* polymorphisms with BMD phenotypes (all *P* > 0.05).

## Discussion

In this meta-analysis, we investigated the association of the *LRP5* A1330V, V667M and Q89R polymorphisms with BMD. The *LRP5* gene is a known locus associated with BMD in several genome-wide association studies (GWASs). Although early studies were carried out in Caucasians [6-8], the 11q13 locus (*LRP5*) was also reported to be associated with BMD in replication study in the East-Asians [43]. There were meta-analyses on the association between the *LRP5* gene and BMD [44,45], however, both studies only examined the influence of the A1330V polymorphism on BMD. Moreover, limited studies were included in both meta-analyses. In the present study, more unbiased new articles with a larger sample size could provide new insight into the underlying relationship between the *LRP5* gene and BMD. This is the most comprehensive meta-analysis focused on the associations of the A1330V, Val667Met and Q89R polymorphisms with BMD, which involved 19 studies with 25773 subjects in total. Our results suggested that the A1330V and Val667Met polymorphisms were related to LS and FN BMDs, and that the Q89R polymorphism was associated with FN BMD.

There have been several studies reported that the A1330V polymorphism is associated with BMD, and that individuals with the AV/VV or VV genotype have lower BMD than those with the AA genotype. Our data also supported a significant association between this SNP and BMD at LS and FN among Caucasian populations, which is consistent with previous GWAS studies [7]. However, we did not detect any association of this polymorphism with FN BMD among men or Asians. It has been observed that the relative distribution of the A1330V genotypes varied remarkably between genders as well as among different populations. For example, the AA genotype was 46%-73.5% in Asian women [23,25], however, much higher (73%) in Dutch women [22]. Among Caucasian populations, the AA genotype was detected in 56% of Dutch men [22], 91% of Finnish men [20], and 76% of Danish men [21]. In addition, there were statistically significant differences in A1330-allele frequencies with regard to gender in our study (*P* < 0.05). Possible explanation for the gender-related differences might be that sex-specific hormones (such as androgens and estrogens) were involved in the regulation of *LRP5*. LRP5, with its close homolog LRP6, functions as a cell membrane co-receptor for Wnt proteins in the canonical Wnt signaling pathway. Interestingly, a previous study reported the direct evidence of cross-talk between Wnt and estrogen signaling pathways via functional interaction between β-catenin and estrogen receptor (ER)-α*in vivo* [46]. The results from another more recent article also suggested the osteoporotic phenotype of ER knock-in (ERα^−/NERKI^) mice may involve the suppression of lymphoid enhancer factor-1 (Lef1)-mediated Wnt signaling [47]. Moreover, the study by Noh et al. [48] showed the *Lef1* haploinsufficient (*Lef1* ^*+/-*^) mice display low bone mass phenotype in a gender- and age- specific manner. The findings of these functional studies, combined with our results, indicated that the effect of the *LRP5* gene on BMD might be affected by gender.

For the analysis of V667M, our study consisted of 7 articles which were all using Caucasian subjects, while the analysis for the Q89R polymorphism included two studies both performed in Asian population. Significant association was observed between the V667M polymorphism and BMDs at LS and FN, which agreed with previous studies [21,24,31,32,36,40]. We also found that subjects with the Q89R QQ genotype tended to have high BMD than those with QR/RR genotypes. Our results appeared to be further supported by the positions at which these polymorphisms were situated in the *LRP5* gene. LRP5 is a single pass membrane receptor whose extracellular protein contains four domains resembling a propeller with six blades containing YWTD spacer repeats followed by an epidermal growth factor (EGF)-like module, and an LDL receptor-like ligand-binding domain [49,50]. The Q89R polymorphism is located in exon 2, which encodes the first of four propellers, while V667M is localized at the top of the third propeller module. Although the precise function of each region is uncertain, the four propellers are of structural importance. It has been suggested that mutation in the first propeller region can alter the local hydrophobic environment, thus possibly affecting the interaction of LRP5 with other proteins [18]. In addition, the A1330V polymorphism is in exon 18 encoding the LDL receptor-like domain. It has been reported that a number of mutations in the LDL receptor propeller module can cause familial hypercholesterolemia [49], confirming that this domain is important for protein function. These findings provide evidence that all these polymorphisms might have the ability to alter the LRP5 activity, which in turn had an impact on BMD. But the question that arises is to what extent each SNP would exert influence on BMD.

The Q89R and A1330V polymorphisms were in linkage disequilibrium in Korean and Chinese populations [25,26], but not among a European population [51]. Strong linkage disequilibrium was also observed between the SNPs V667M and A1330V [22,34,52]. In the study by van Meurs et al. [22], the haplotype analysis indicated that the A1330V polymorphism was probably driving the association with BMD, because two haplotypes containing the 1330V variant showed an association with low BMD and other bone-related endpoints, whereas the other haplotypes did not. It has been found that another SNP at the intron 17 (IVS17-1677C>A) in the *LRP5* gene was related to total body BMD in Japanese subjects [53]. Further study by the same team showed that the IVS17-1677C>A SNP and A1330V were in strong linkage disequilibrium [23]. These data suggested a possibility that the A1330V polymorphism or other polymorphisms in linkage disequilibrium with it, which have not been identified yet, might be the main driving force behind the association with BMD. However, replications of this association in different ethnic populations as well as *in vitro* functional studies are warrant to validate the hypothesis.

In interpreting the results, some limitations of this meta-analysis should be addressed. First, our results were based on unadjusted estimates, while a more precise analysis should be conducted if all individual raw data were available, which would allow for the adjustment by other covariates including age, body height, body weight, and exercises habits. Second, the analysis for V667M was totally based on data of Caucasian subjects, while the analysis for Q89R was based on two studies performed in Asians. Notably, the V667M SNP was not polymorphic in the Hong Kong population [32], and the Q89R polymorphism was very rare in Caucasians [51,54]. Therefore, additional studies in different populations focused on other loci which are in LD with these variations are needed to further validate ethnic difference in their effects on BMD. Third, the effects of gene-gene or gene-environment interactions were not addressed in this meta-analysis. However, most studies did not provide the detailed information, which impeded us for further analysis.

In conclusion, the present study is the most comprehensive meta-analysis investigating the associations of the *LRP5* polymorphisms with BMD. And our data demonstrated that the A1330V and V667M polymorphisms were significantly associated with BMDs at LS and FN, while the Q89R polymorphism was significantly associated with FN BMD. For future association studies, more accurate phenotype and genotype data, detailed individual information, larger sample size of different ethnic populations and standard statistical methods will be needed. Moreover, the interactions between gene-gene and gene-environment should also be evaluated. 

## Supporting Information

Checklist S1 (DOC)

Table S1 **The process of study selection for the meta-analysis.**
(DOCX)

Figure S1 **Funnel plot for LS BMD between A1330V AA and AV/VV genotypes.**
(TIF)

Figure S2 **Funnel plot for FN BMD between A1330V AA and AV/VV genotypes.**
(TIF)

Figure S3 **Funnel plot for LS BMD between V667M VV and VM/MM genotypes.**
(TIF)

Figure S4 **Funnel plot for FN BMD between V667M VV and VM/MM genotypes.**
(TIF)

Figure S5 **Funnel plot for FN BMD between Q89R QQ and QR/RR genotypes.**
(TIF)
