# Supplementary material for: Genetic Analysis of the Relationship between Bone Mineral Density and Low-Density Lipoprotein Receptor-Related Protein 5 Gene Polymorphisms
Source: PLoS One. 2013 Dec 23;8(12):e85052. doi: 10.1371/journal.pone.0085052 (PMC3871666; doi:10.1371/journal.pone.0085052)
Supplement: Table S1 — The process of study selection for the meta-analysis. (DOCX) [file pone.0085052.s002.docx]

Supplementary Table S1. The process of study selection for the meta-analysis.

| Study | Included or excluded status | Exclusion classification |
| --- | --- | --- |
| García-Ibarbia C et al., Gene. 2013 | Excluded | Ⅱ |
| Méndez JP et al., Gene. 2013 | Excluded | Ⅱ |
| Galora S et al., J Vasc Surg. 2013 | Excluded | Ⅱ |
| Boudin E et al., Bone. 2013 | Excluded | Ⅱ |
| Falcón-Ramírez E et al., Mol Biol Rep. 2013 | Excluded | Ⅱ |
| Massart F et al., Joint Bone Spine. 2013 | Included | NA |
| de Rooy DP et al., Ann Rheum Dis. 2013 | Excluded | Ⅱ |
| Estrada K et al., Nat Genet. 2012 | Excluded | Ⅴ |
| Markatseli AE et al., Maturitas. 2011 | Included | NA |
| Urano T, Nihon Rinsho. 2011 | Excluded | Ⅱ |
| Kim H et al., Menopause. 2011 | Excluded | Ⅱ |
| Korvala J et al., BMC Genet. 2010 | Excluded | Ⅴ |
| Yu JB et al., Acta Pharmacol Sin. 2010 | Included | NA |
| Styrkarsdottir U et al., PLoS One. 2010 | Excluded | Ⅱ |
| Riancho JA et al., Eur J Endocrinol. 2011 | Included | NA |
| Stathopoulou MG et al., J Am Diet Assoc. 2010 | Included | NA |
| Lee DY et al., Menopause. 2010 | Excluded | Ⅱ |
| Liu JM et al., J Clin Endocrinol Metab. 2010 | Excluded | Ⅴ |
| Ichikawa S et al., J Bone Miner Res. 2010 | Excluded | Ⅳ |
| Jiang XY et al., Exp Clin Endocrinol Diabetes. 2010 | Included | NA |
| Li WF et al., Hum Genet. 2010 | Excluded | Ⅰ |
| Piters E et al., Calcif Tissue Int. 2010 | Excluded | Ⅱ |
| Marques-Pinheiro A et al., Joint Bone Spine. 2010 | Excluded | Ⅱ |
| Mencej-Bedrac S et al., Calcif Tissue Int. 2009 | Included | NA |
| Richards JB et al., Ann Intern Med. 2009 | Excluded | Ⅳ |
| Rivadeneira F et al., Nat Genet. 2009 | Excluded | Ⅳ |
| Utriainen P et al., J Clin Endocrinol Metab. 2009 | Excluded | Ⅱ |
| Urano T et al., Endocr J. 2009 | Excluded | Ⅳ |
| Giampietro PF et al., Osteoporos Int. 2010 | Excluded | Ⅰ |
| Velasco J et al., Osteoporos Int. 2010 | Excluded | Ⅱ |
| Nissen N et al., Calcif Tissue Int. 2009 | Excluded | Ⅴ |
| Kruk M et al., Calcif Tissue Int. 2009 | Included | NA |
| Greene R et al., Pharmgenomics Pers Med. 2009 | Excluded | Ⅱ |
| Ferrari S et al., Best Pract Res Clin Endocrinol Metab. 2008 | Excluded | Ⅰ |
| Furuya T et al., Mod Rheumatol. 2009 | Excluded | Ⅴ |
| Yerges LM et al., J Bone Miner Res. 2009 | Excluded | Ⅱ |
| Agueda L et al., J Bone Miner Res. 2008 | Excluded | Ⅳ |
| Richards JB et al., Lancet. 2008 | Excluded | Ⅳ |
| Giroux S et al., Osteoporos Int. 2008 | Included | NA |
| van Meurs JB et al., JAMA. 2008 | Excluded | Ⅴ |
| Urano T, Nihon Rinsho. 2007 | Excluded | Ⅰ |
| Cheung CL et al., Hum Hered. 2008 | Excluded | Ⅱ |
| Rodríguez-Bores L et al., World J Gastroenterol. 2007 | Excluded | Ⅰ |
| Brixen K et al., Calcif Tissue Int. 2007 | Included | NA |
| Grundberg E et al., Osteoporos Int. 2008 | Included | NA |
| Sims AM et al., J Bone Miner Res. 2008 | Excluded | Ⅱ |
| Kiel DP et al., BMC Med Genet. 2007 | Excluded | Ⅲ |
| Koay MA et al., Calcif Tissue Int. 2007 | Excluded | Ⅱ |
| Giroux S et al., Bone. 2007 | Included | NA |
| Ezura Y et al., Bone. 2007 | Included | NA |
| Xiong DH et al., J Bone Miner Res. 2007 | Excluded | Ⅱ |
| Saarinen A et al., Bone. 2007 | Included | NA |
| Urano T et al., Spine (Phila Pa 1976). 2007 | Excluded | Ⅱ |
| Kiel DP et al., Bone. 2007 | Excluded | Ⅲ |
| Zofková I et al., Physiol Res. 2007 | Excluded | Ⅱ |
| Li Y et al., Wei Sheng Yan Jiu. 2006 | Excluded | Ⅱ |
| Norek A et al., Med Wieku Rozwoj. 2006 | Excluded | Ⅱ |
| Xiong DH et al., J Bone Miner Res. 2006 | Excluded | Ⅱ |
| Ralston SH et al., Genes Dev. 2006 | Excluded | Ⅰ |
| Zajickova K et al., Clin Chem Lab Med. 2006 | Excluded | Ⅱ |
| Guo J et al., Bone. 2007 | Excluded | Ⅱ |
| Cheung WM et al., Bone. 2006 | Excluded | Ⅱ |
| Lau HH et al., J Bone Miner Metab. 2006 | Excluded | Ⅱ |
| Inoue S, Nihon Ronen Igakkai Zasshi. 2006 | Excluded | Ⅱ |
| Hosoi T, Nihon Ronen Igakkai Zasshi. 2005 | Excluded | Ⅱ |
| Urano T, Clin Calcium. 2006 | Excluded | Ⅰ |
| van Meurs JB et al., J Bone Miner Res. 2006 | Included | NA |
| Crabbe P et al., J Bone Miner Res. 2005 | Excluded | Ⅴ |
| Ferrari SL et al., Bone. 2005 | Excluded | Ⅱ |
| Zhang ZL et al., Acta Pharmacol Sin. 2005 | Included | NA |
| Smith AJ et al., Osteoarthritis Cartilage. 2005 | Excluded | Ⅱ |
| Ferrari SL et al., Mol Aspects Med. 2005 | Excluded | Ⅰ |
| Davies JH et al., Arch Dis Child. 2005 | Excluded | Ⅰ |
| Lau HH et al., Bone. 2005 | Excluded | Ⅱ |
| Bollerslev J et al., Bone | Excluded | Ⅱ |
| Ferrari SL et al., Curr Opin Lipidol. 2005 | Excluded | Ⅰ |
| Koay MA et al., Trends Mol Med. 2005 | Excluded | Ⅰ |
| Koller DL et al., J Bone Miner Res. 2005 | Included | NA |
| Johnson ML et al., J Bone Miner Res. 2004 | Excluded | Ⅰ |
| Koay MA et al., J Bone Miner Res. 2004 | Excluded | Ⅱ |
| Urano T et al., J Bone Miner Metab. 2004 | Excluded | Ⅱ |
| Koh JM et al., J Korean Med Sci. 2004 | Included | NA |
| Ferrari SL et al., Am J Hum Genet. 2004 | Excluded | Ⅱ |
| Mizuguchi T et al., J Hum Genet. 2004 | Included | NA |
| Takács I et al., Orv Hetil. 2003. | Excluded | Ⅰ |
| Van Wesenbeeck L et al., Am J Hum Genet. 2003 | Excluded | Ⅱ |

Class Ⅰ: Reviews or editorials

Class Ⅱ: Studies not meet the purpose of present meta-analysis

Class Ⅲ: Overlapping data

Class Ⅳ: Insufficient data

Class Ⅴ: No desirable outcome
