# Supplementary figures and images for: Genetic Analysis of the Relationship between Bone Mineral Density and Low-Density Lipoprotein Receptor-Related Protein 5 Gene Polymorphisms
Source: PLoS One. 2013 Dec 23;8(12):e85052. doi: 10.1371/journal.pone.0085052 (PMC3871666; doi:10.1371/journal.pone.0085052)

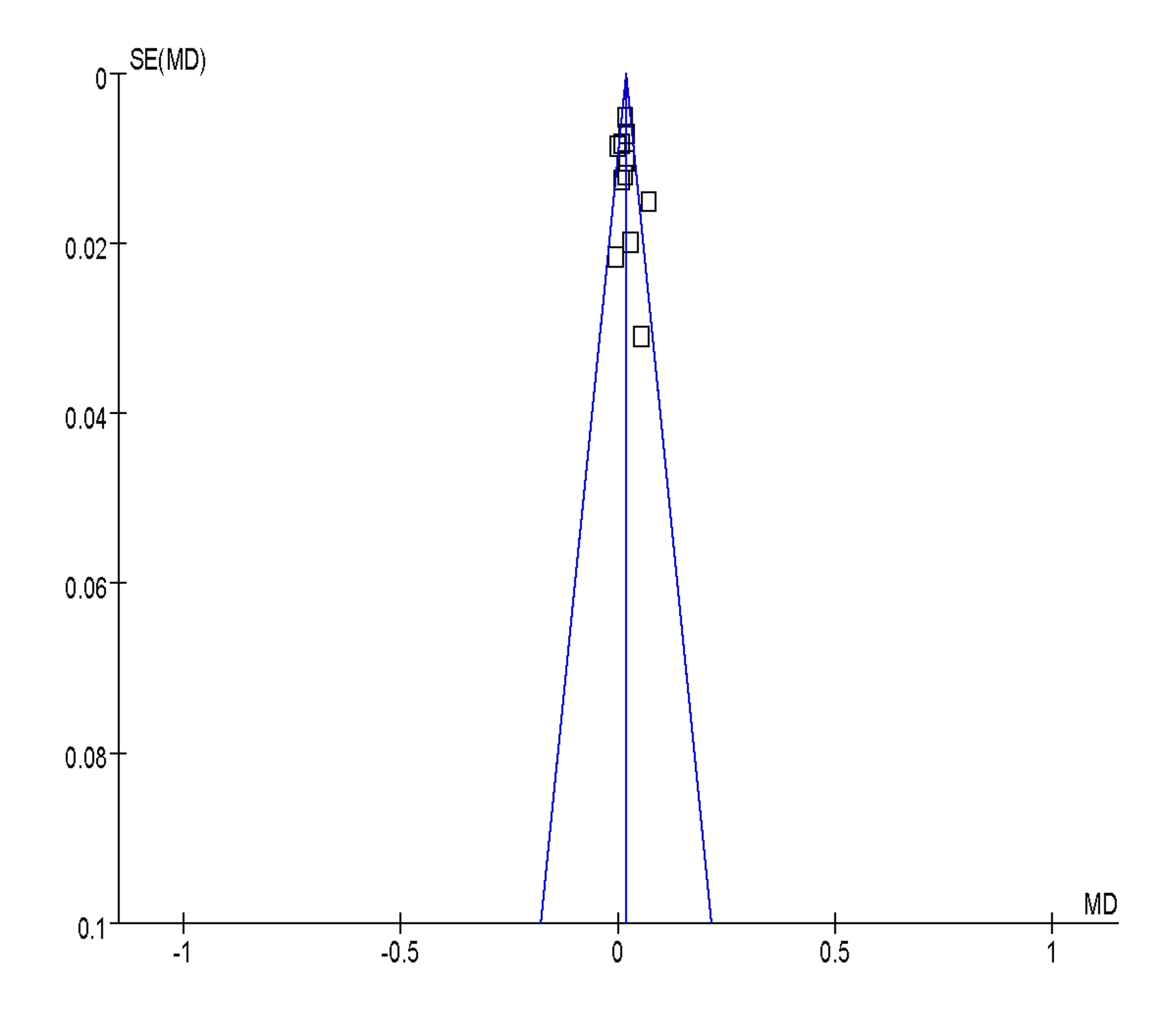

Supplement: Figure S1 — Funnel plot for LS BMD between A1330V AA and AV/VV genotypes. (TIF) [file pone.0085052.s003.tif]

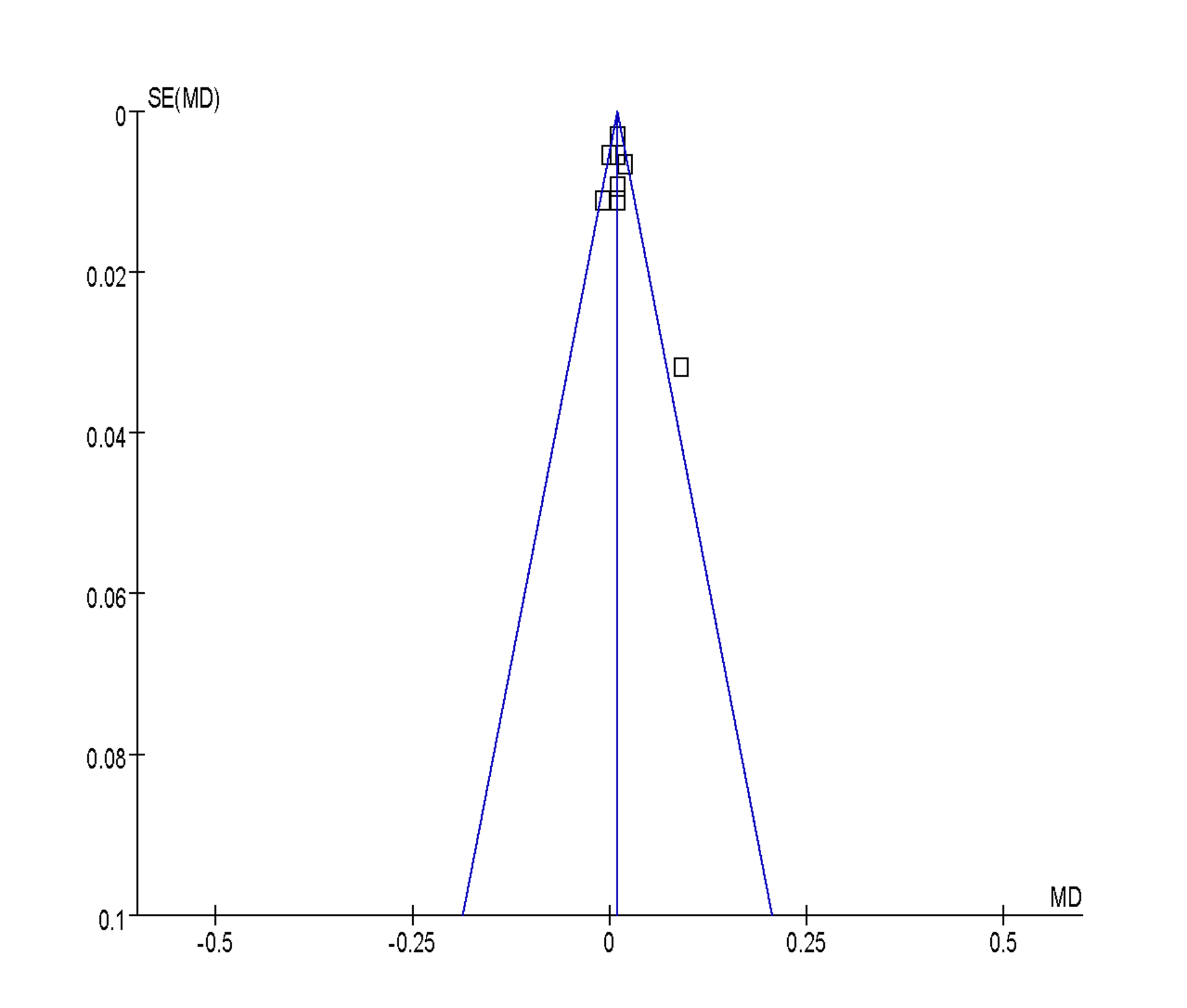

Supplement: Figure S2 — Funnel plot for FN BMD between A1330V AA and AV/VV genotypes. (TIF) [file pone.0085052.s004.tif]

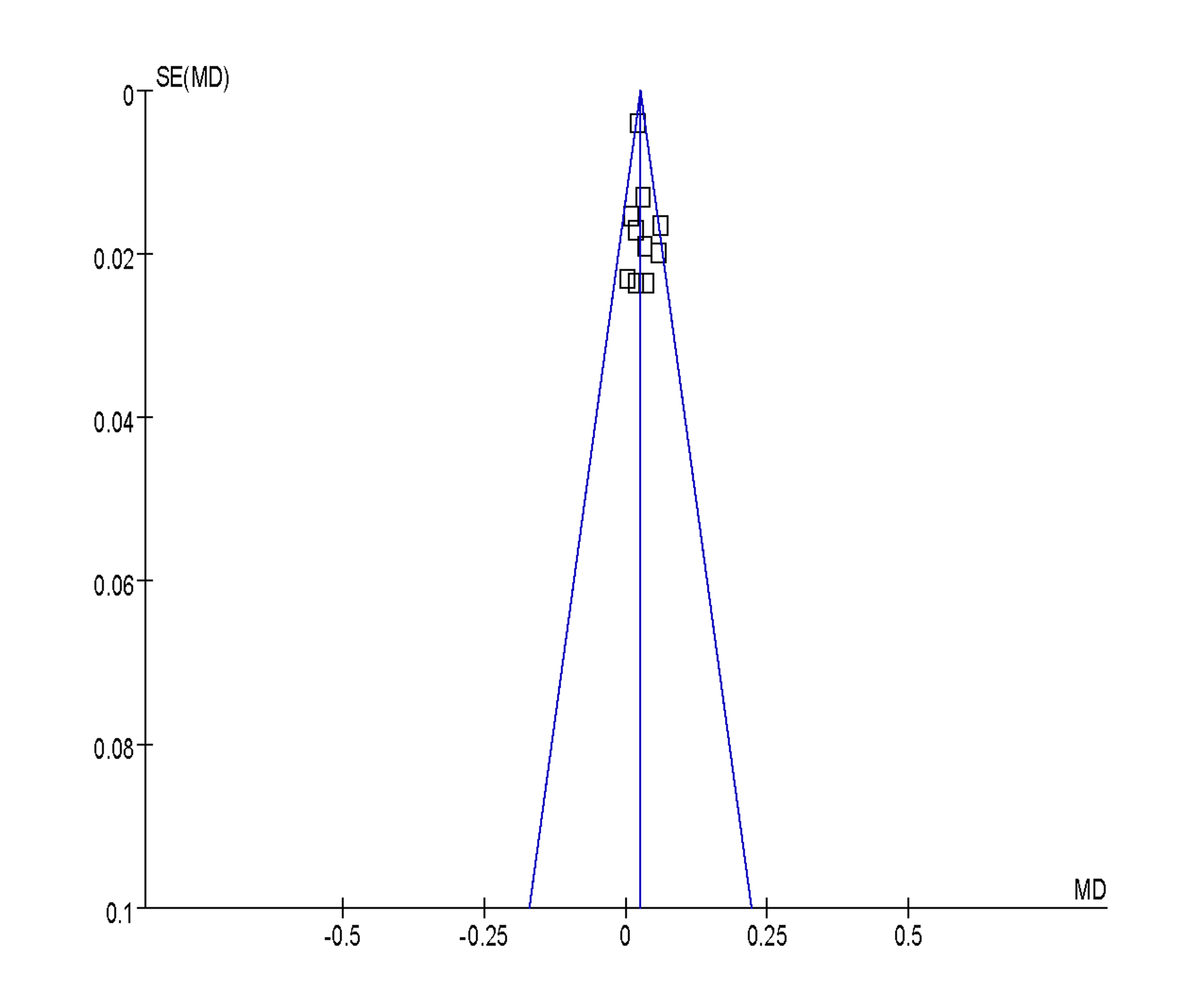

Supplement: Figure S3 — Funnel plot for LS BMD between V667M VV and VM/MM genotypes. (TIF) [file pone.0085052.s005.tif]

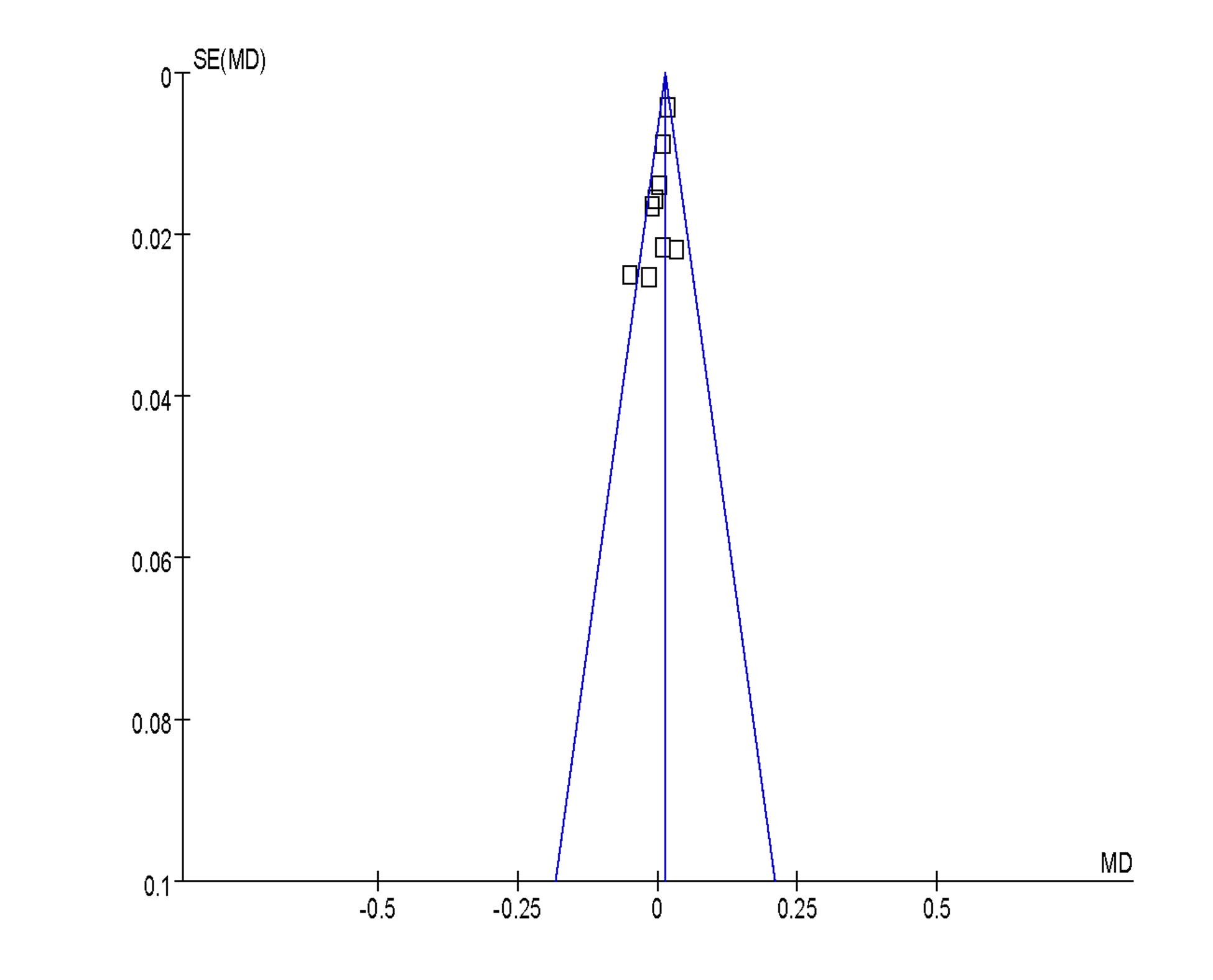

Supplement: Figure S4 — Funnel plot for FN BMD between V667M VV and VM/MM genotypes. (TIF) [file pone.0085052.s006.tif]

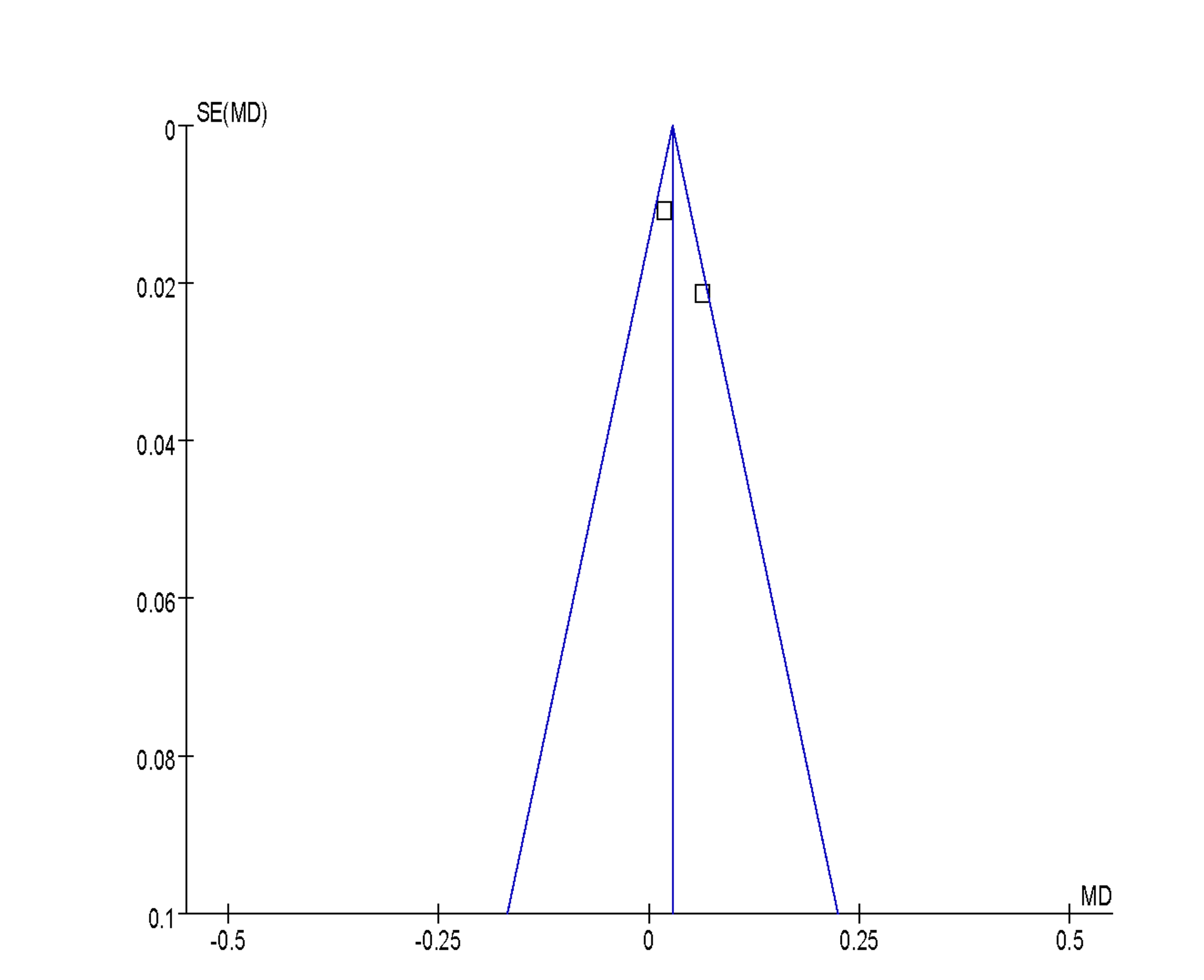

Supplement: Figure S5 — Funnel plot for FN BMD between Q89R QQ and QR/RR genotypes. (TIF) [file pone.0085052.s007.tif]
